# Evaluating for learning and sustainability (ELS) framework: a realist synthesis

**DOI:** 10.1186/s12913-025-12743-4

**Published:** 2025-05-13

**Authors:** Marissa Bird, Maura MacPhee, James Shaw, Walter P. Wodchis, Lianne Jeffs, Tujuanna Austin, Frances Bruno, Balpreet Panesar, Élizabeth Côté Boileau, Robert J. Reid, Carolyn Steele Gray

**Affiliations:** 1 Institute for Better Health, Trillium Health Partners, Mississauga, ON Canada; 2 Institute of Health Policy, Management and Evaluation, University of Toronto, Toronto, ON Canada; 3 University of British Columbia School of Nursing, Vancouver, BC Canada; 4 Department of Physical Therapy, Temerty Faculty of Medicine, University of Toronto, Toronto, ON Canada; 5 Sinai Health, Toronto, ON Canada; 6 Department of Health Management, Evaluation and Policy, School of Public Health, University of Montreal, Montreal, QC Canada

**Keywords:** Innovation, Evaluation, Sustainability, Learning, Learning health systems

## Abstract

**Background:**

Learning Health Systems (LHS), in which continuous and equitable improvements support optimization of healthcare practices, outcomes, experience, and costs, offer enormous potential for health system transformation. Within the LHS model, evaluation of health innovations assists in question identification, data collection, and targeted action, which facilitates continuous improvement. Evaluation that catalyzes learning may contribute to health innovation implementation, refinement, and sustainability, however, there is little consensus as to why certain evaluations support learning, while others impede it.

**Methods:**

Embedded in the implementation science literature, we conducted a realist synthesis to understand evaluative contextual factors and underlying mechanisms that best support health system learning and sustainable implementation of innovations. We sought to understand whether evaluations can ‘work’ to support learning and sustainability, in which contexts, for whom, and why. Working with an Expert Committee comprised of leaders in evaluation, innovation, sustainability, and realist methodology, we followed a five-stage process of: 1. Scoping the Review, 2. Building Theories, 3. Identifying the Evidence, 4. Evidence Selection and Appraisal, and 5. Data Extraction and Synthesis. Our Review Team and Expert Committee participated in iterative cycles of results interpretation and feedback.

**Results:**

Our synthesis includes 60 articles capturing the mechanisms and contextual factors driving learning and sustainability through evaluation. We found that evaluations that support learning and sustainability incorporate favourable organizational preconditions and focus on implementing rapid cyclical feedback loops that contribute to a culture of innovation and evaluation sustainability. Our findings have been organized into 6 Context-Mechanism-Outcome Configurations (CMOCs): 1. Embracing Risk & Failure; 2. Increasing Capacity for Evaluation; 3. Co-Producing Evaluation; 4. Implementing Learning Feedback Loops; 5. Creating Sustainability Culture; and 6. Becoming a Learning Organization. We have also translated findings into a series of Action Strategies for evaluation implementation to support health systems learning and sustainability.

**Conclusions:**

We identified key contextual factors and underlying mechanisms that make evaluations ‘work’ (or ‘not work’) to support learning and sustainability. Findings support the operationalization of LHS by translating CMOCs into Action Strategies for those tasked with completing evaluations with a view toward health system learning and innovation sustainability.

**Supplementary Information:**

The online version contains supplementary material available at 10.1186/s12913-025-12743-4.

## Introduction

The global COVID- 19 pandemic brought to light intractable challenges that health systems have grappled with for years, throwing into sharp relief the scale and severity of hospital overcrowding, surgical backlogs, and workforce burnout, among other challenges [1–4]. In the coming years, escalating healthcare acuity, growing service needs, and widening inequities will continue to pose a threat to healthcare systems globally [5, 6], prompting healthcare systems to begin to shift attention to the future [7], seeking to understand and articulate how they will transform the care they provide for the populations they serve. As healthcare systems contemplate new approaches to improving health and delivering healthcare that prioritize both the needs of patients and populations, it is essential to understand how we can approach complex challenges differently by generating, learning from, and sustaining health innovations in order to realize the promise of healthcare system transformation.

Health innovations are novel, contextually-situated approaches which hold promise to accelerate and sustain positive health impacts by responding to health needs and preferences (see Table 1 for terminology used throughout this manuscript) [8]. Health innovations can and should be driven by the needs of end-users of the healthcare system, including patients, families, and community members [9], as well as clinicians, researchers, and health system leaders [10]. While generating scientific and healthcare delivery innovations is a vital activity for health system improvement [10–12], equally important is the ability to sustain health innovations through robust implementation and evaluation processes [13–15]. Learning Health Systems (LHS) offer an approach to moving knowledge to action by engaging in continuous healthcare system improvement via innovation– converting evidence to knowledge, applying that knowledge to influence innovation performance, and generating new evidence through observation of innovation performance changes [16, 17]. While both LHS and other healthcare improvement methodologies such as continuous quality improvement utilize cyclical data-driven processes to improve care, LHS focus on creating scalable systems that address population and person-centred health and using rigorous methods to do so [17]. The essential function of evaluation within the LHS cycle is its capacity to catalyze systems learning from improvement efforts, supporting a trajectory of knowledge to action. Thus, embedded evaluation drives learning and innovation sustainability within an LHS by offering a consistent cycle of feedback with which to ensure continued innovation fit for context [18] by revealing areas where adaptation and tailoring could help to improve innovation fit.

**Table 1:** Terminology and definitions

Term	Definition
Context	The backdrop of conditions in which interventions are implemented [19]. These conditions include circumstances which trigger and/or modify mechanisms (e.g., historical events, cultural norms, existing social networks, funding sources, participant characteristics, and opportunities or constraints offered by interventions) [19].
Evaluation	Efforts to assess and understand multiple aspects of LHS performance [20], including the merit, impact, enactment, and experience of those interacting with health innovations. Evaluations can be conducted at any stage of the innovation lifecycle (e.g., formative, developmental, summative) and use a wide variety of methods to generate insight.
Evaluator	An individual tasked with planning and conducting evaluation tasks.
Health Innovation	New or improved solutions with the potential to accelerate positive health impact, including health outcomes, experience, costs, and equity, among other outcomes [8].
Institutionalization	Establishing a process as a norm or convention in an organization.
Leader	An individual within a health organization that sets direction, influences others, and is responsible for managing change [21].
Learning Health System	A system which aligns science, informatics, incentives, and culture to target continuous improvement, innovation, and equity [22].
Mechanism	Causal forces, including underlying entities, processes, or structures which operate in particular contexts to generate outcomes [19].
Outcome	The results of mechanisms operating in particular contexts.
Program Theory	An explanation of how and why a class of intervention ‘works’ to generate outcomes of interest [23].
Staff	Health professionals and other individuals that work within the healthcare system to operationalize and use health innovations.
Sustainability	The stability and endurance of engrained change of an innovation within a health system to become the “new normal” [24].

**Fig. 1:**
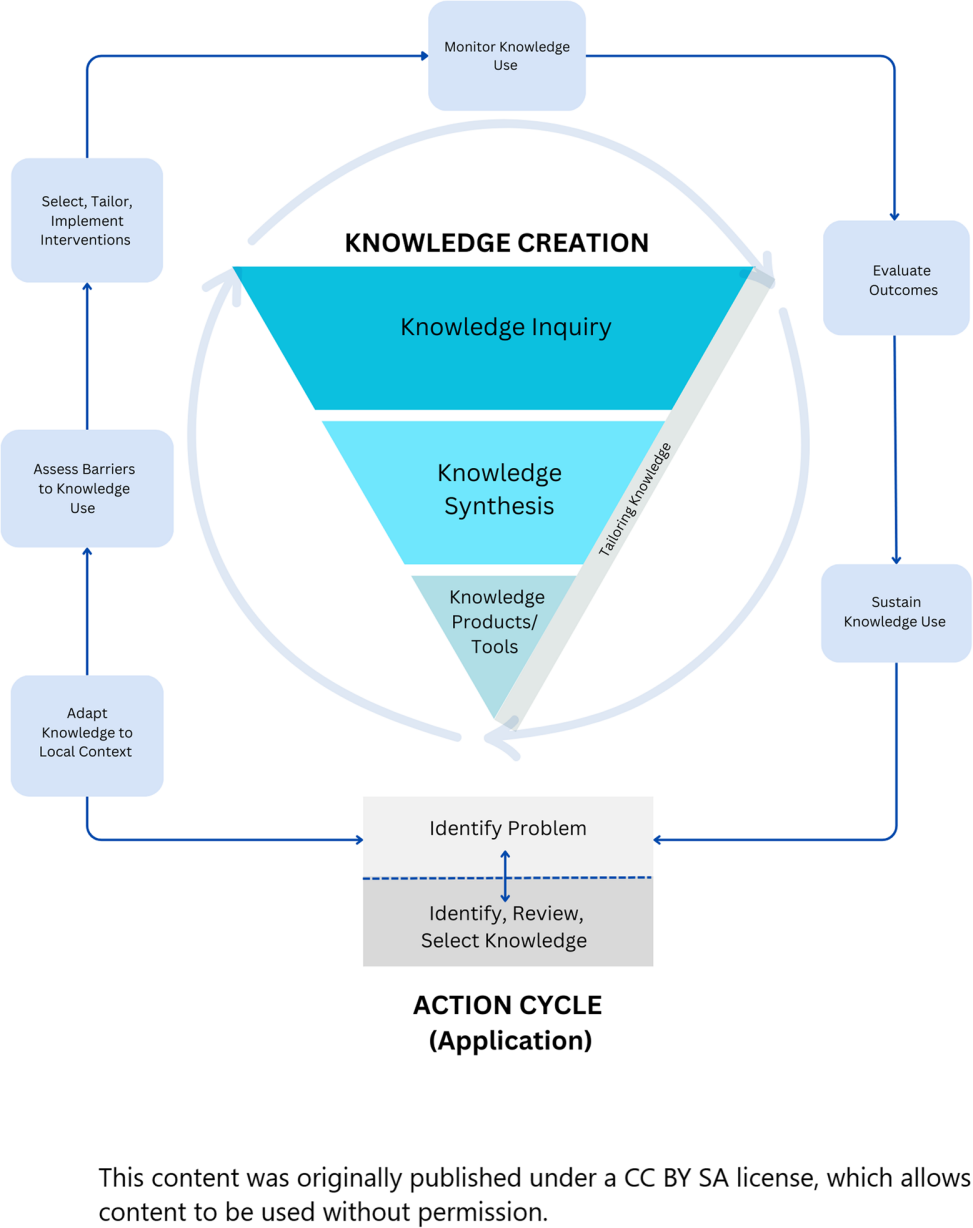
The knowledge to action framework [15]

The conceptual link between evaluation of health innovations and the sustainability of those innovations is supported empirically [25] and conceptually [18, 26] and is depicted by a directional arrow in the Knowledge to Action (K2 A) Framework [15] (Fig. 1). The K2 A framework is one example of an implementation framework that illustrates the link between evaluation and sustainability at the end of the innovation cycle. Unfortunately, this link is often overlooked, impeding the ability of evaluation to enhance sustainability throughout the innovation process. Additionally, many implementation science frameworks indicate a link between evaluation and sustainability, including the K2 A [15], the Exploration, Preparation, Implementation, and Sustainment (EPIS) framework [27], and Normalization Process Theory [28, 29]. However, the quality of the relationship between these two stages is as yet underexplored in terms of how evaluation can contribute to learning and innovation sustainability, and in which circumstances this takes place. As the purpose of implementation science is to move knowledge into practice, an understanding of how learning and sustainability happen with respect to evaluation, and under which conditions these processes take place, can further this aim. The purpose of this realist synthesis is to explore the quality of evaluations of learning health systems to assess which components of evaluations are necessary to generate learning and improve innovation sustainability. Findings will add to the LHS and implementation literature by specifying the necessary approaches for implementing evaluations that support learning and sustainability. In this synthesis, the evaluation of health innovations is the ‘intervention’ under study. Our realist synthesis question is: What types of evaluations work to promote LHS learning and health innovation sustainability, for whom do they work, under which circumstances, and why?”.

## Methods

We conducted a five-stage realist synthesis in line with RAMESES guidelines (see Additional file 1) [23, 30]. Realist synthesis is a complexity-compatible review method that examines interventions to understand “what works, for whom, under which circumstances, and why?” [31]. In realist terms, variations in outcomes (O) can be attributed to the differing contexts (C) into which interventions are implemented, and the resultant mechanisms (M) that are activated due to contextual factors such as time, place, people, and social and political structures [32, 33]. The working hypothesis of this paper is that different evaluative approaches, measurement, reporting, and data use techniques, and selection of indicators and outcomes work to support LHS learning and innovation sustainability, while others do not.

Our previously published protocol [32] contains additional details of our review methods. Our team undertook the following stages in this review: 1. Scoping the Review, 2. Building Theories, 3. Identifying the Evidence, 4. Evidence Selection and Appraisal, and 5. Data Extraction and Synthesis.

### Scoping the review and building theories

**Fig. 2:**
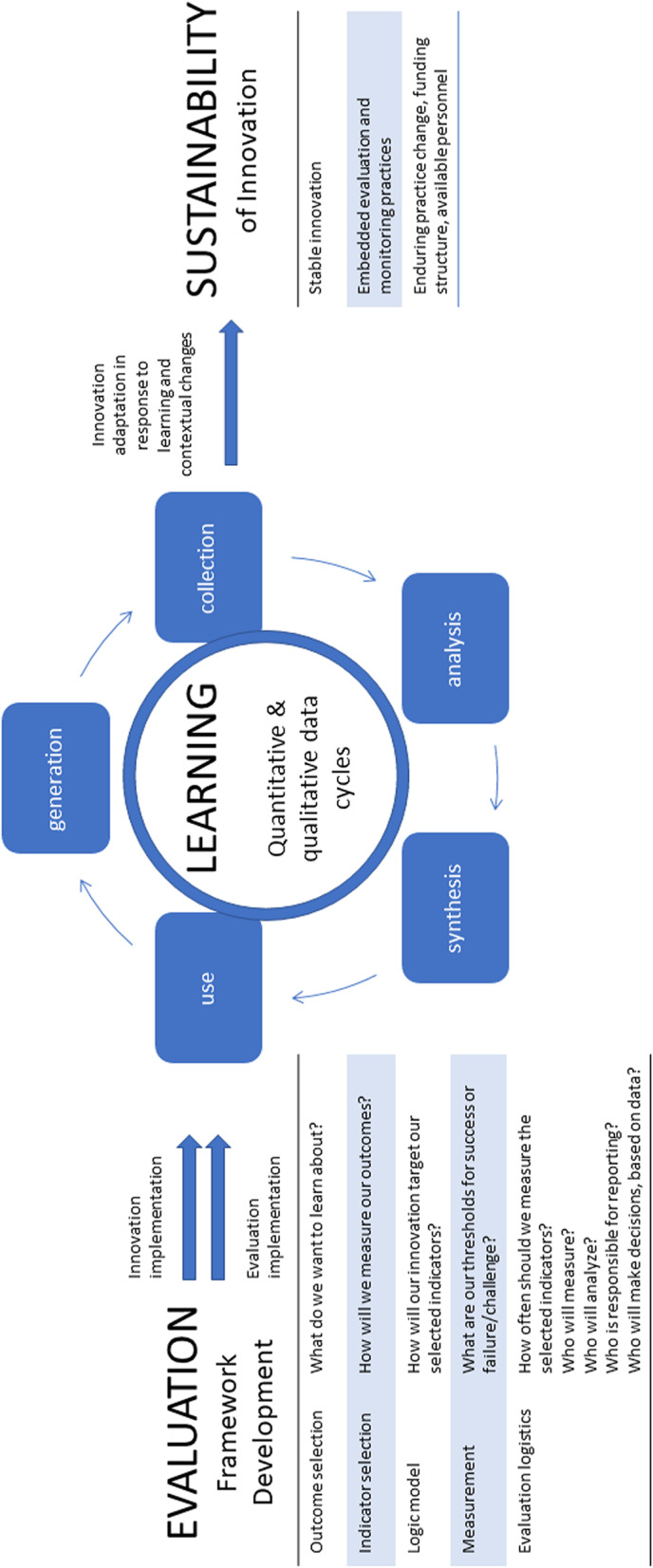
Initial program theory

Our group divided into two teams – an Expert Consultation Committee (co-authors CSG, ÉCB, JS, LJ, MB, MM, RJR, WPW) and a Review Team (BP, FB, TA). The Expert Consultation Committee was comprised of interdisciplinary subject matter experts in fields related to the scope of this review, such as learning health systems, evaluation, health innovation, sustainability, as well as realist methodology. Their role was to provide ongoing conceptual and methodological feedback throughout the review process to shape the research questions, literature review scope, and analysis process. The Review Team was comprised of health services research doctoral students and a research associate who, alongside the lead author, undertook the majority of the searching, screening, and the initial stages of data analysis. Initially, these teams worked together to combine their collective wisdom, using twenty-five foundational articles from the fields of LHS, evaluation, and health innovation sustainability, to formulate an Initial Program Theory of the conceptual link between evaluation, learning, and sustainability (See Fig. 2). We hypothesized that evaluation of an innovation begins with the development of an evaluation framework that includes the components on the left side of Fig. 2. The learning cycle from evaluation is based on the collection, analysis, synthesis, use and generation of evidence that must be adapted to suit local, contextual factors that promote sustainable uptake of new practices and a new evaluation-learning-sustainability cycle.

### Identifying the evidence

**Fig. 3:**
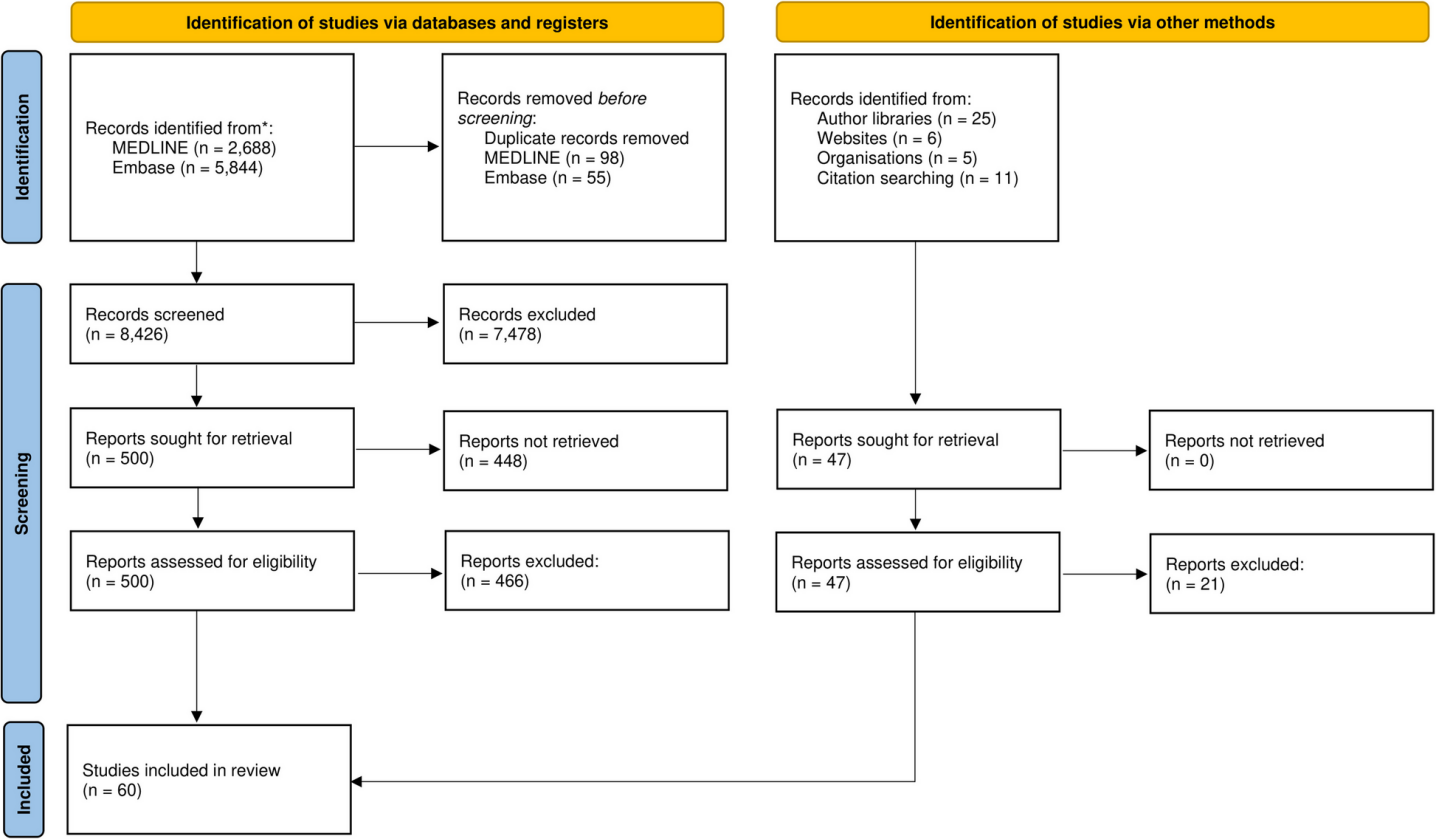
PRISMA flow diagram

We conducted initial background searches to get a ‘feel’ for the evidence base [31], before building progressively focused searches aimed at illuminating the contexts and mechanisms associated with the link between evaluation, learning, and LHS sustainability. We began our primary search using the MEDLINE (National Library of Medicine) and Embase (Elsevier) databases using a combination of MeSH terms and key words conceptually centred around ‘evaluation’, ‘learning’, and ‘sustainability’, and ‘healthcare’. We limited our search to 2013–2023 to account for the growth of interest in the sustainability of health innovations over the last decade. A sample MEDLINE search can be found in Additional file 2. Our PRISMA Diagram (Fig. 3) details of the flow of records through this synthesis.

### Evidence selection and appraisal

Studies were screened and selected through a three-stage process, whereby at the title and abstract level, we included all articles published in English, from 2013 or later, and that related to healthcare innovations, evaluation, and learning and/or sustainability. We excluded articles not written in English, published before 2013, those that were not related to evaluation of healthcare innovations and learning or sustainability, and applied studies that were student-focused or focused on healthcare education (i.e., not in a healthcare setting). The decision to err on the side of inclusion at the title and abstract level stemmed from the realist philosophy that even studies with peripheral relationships to the initial program theory may contain ‘nuggets’ of truth that are useful in developing, iterating, or adjusting working hypotheses [34]. These ‘nuggets’ are often not evident at the title and abstract level and are rather found in the discussion section or lessons learned from published works. At the second level, we included full text articles that, in addition to the criteria above, described a relationship between evaluation and learning or evaluation and sustainability. At the third stage, the first 500 articles were screened for the above criteria, in addition to our assessment of the quality and rigour of the article, and the degree to which it enabled us to further develop our initial program theory. After 500 full text articles were screened at Stage 3, a joint meeting with the Expert Committee and Review Team determined that we had obtained enough high-quality information to move on to data extraction.

### Data extraction and synthesis

Data from 60 included full text articles were extracted into a shared spreadsheet. Three authors (MB, TA, FB) worked together to extract data such as document characteristics (author, year, etc.), central arguments from each article, and realist-informed constructs (e.g., outcomes of interest, contextual factors, possible mechanisms) (see Additional file 1 for operational definitions of extraction criteria). Excerpts of extracted data were then exported to Dedoose [35] qualitative data software for analysis. Excerpts were initially analyzed inductively by grouping concepts into clusters. Next, we examined each cluster to determine where clusters overlapped with concepts from our initial program theory, and where new clusters had to be added or old ones deleted from the initial program theory for congruence. Our Review Team (MB, TA, FB) examined each cluster for relevant contexts, mechanisms, and outcomes to begin to form the basis of our CMOCs. In analyzing articles, we included a search for confirming and disconfirming data to ensure that we were aware of evidence that was at odds with our hypotheses.

**Fig. 4:**
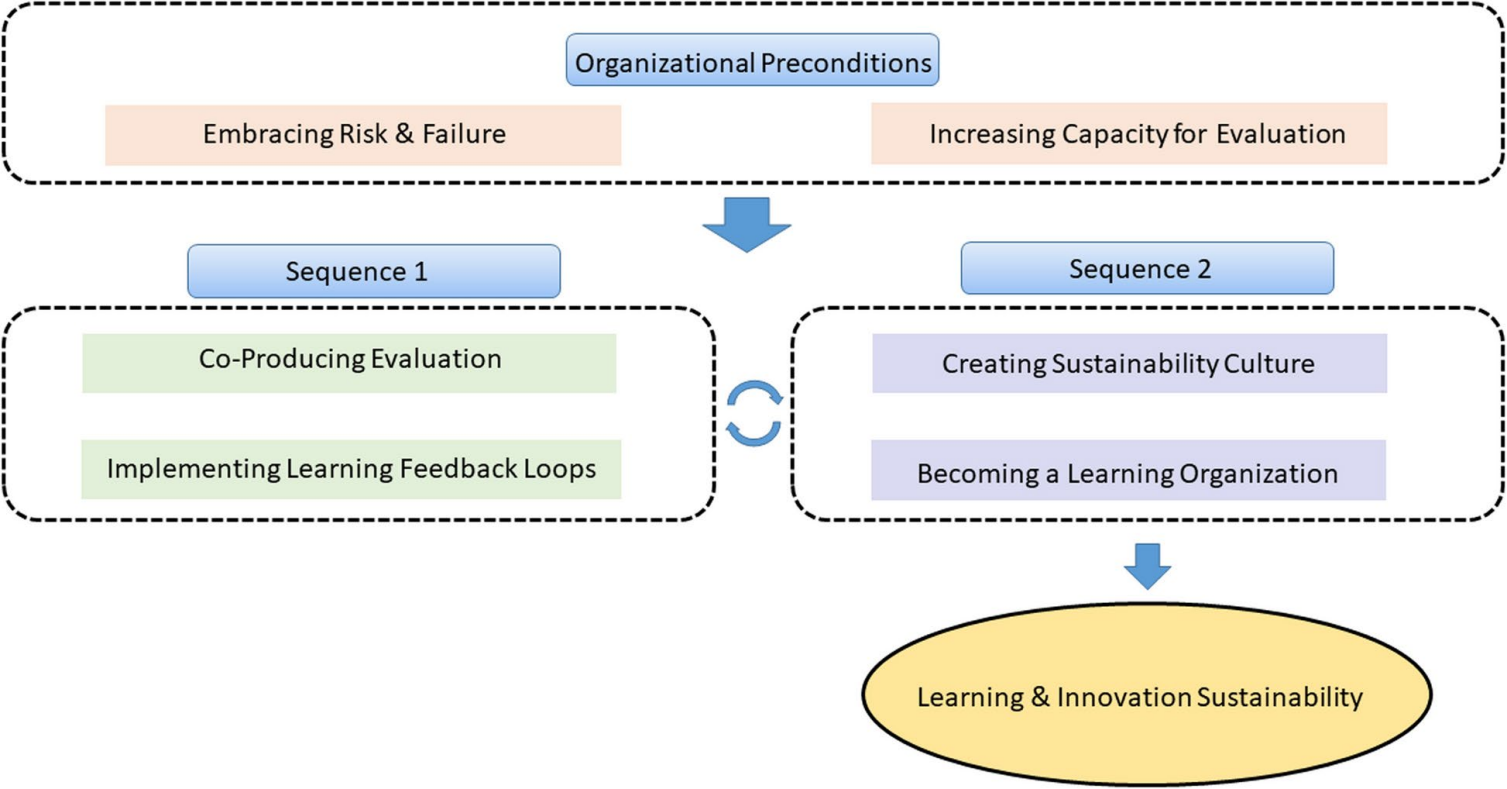
Refined program theory

Next, one author (MB) refined the initial CMOCs using the concept clusters. Initially 14 CMOCs were developed pertaining to six concepts contained within the initial program theory. Excerpts and supporting quotes were filed under each CMOC by one author (MB) while another author (TA) validated CMOCs using an agree/disagree/weak agreement system. CMOCs were refined on the basis of this validation and re-circulated until three authors (MB, TA, FB) achieved consensus as to the comprehensiveness of the CMOCs and the fit of the supporting data within them (Additional file 3). The 14 initial CMOCs were then presented to the Expert Committee for refinement. Through discussion, visual mapping, and asynchronous agreement ratings using bespoke surveys, the initial 14 CMOCs were collapsed into the final six, and the initial program theory was reorganized into the final refined program theory (Fig. 4). The final program theory incorporates the organizational and process-oriented constructs that allow evaluation to promote learning and the sustainability of healthcare innovations.

## Results

Our search identified 8,429 unique results. After title and abstract screening, 547 articles were screened in full text and 487 were excluded to retain 60 articles for inclusion in this synthesis. Data were extracted, analyzed, and synthesized from these articles to refine our initial program theory connecting evaluation to learning and health innovation sustainability. Our refined program theory is detailed below, followed by Table 2 with the CMOCs associated with each stage in the refined program theory.

### Refined program theory

Our results suggest that evaluation can lead to learning and the sustainability of innovations in LHS when evaluation is undertaken in organizations that meet certain preconditions, and where explicit connections exist between distinct sequences of co-production, feedback, and culture change, leading to outcomes of learning and innovation sustainability. The necessary organizational preconditions include embracing risk and failure and working to continuously increase the capacity for evaluation. Under these conditions, evaluations that are co-produced with those involved in the work of innovation, and which drive ongoing and cyclical learning feedback loops can produce ongoing innovation improvements by learning from evaluation data and implementing those learnings to drive decision-making. Outcomes from Sequence 1 activities influence Sequence 2 activities, namely, the creation of a sustainability culture and an effective learning organization that can sustain innovation and evaluation.

### Context-mechanism-outcome configurations

Table 2.

**Table 2:** Context-mechanism-outcome configurations

Organizational Preconditions (CMOC 1 & 2)	
CMOC 1: Embracing Risk & Failure When evaluation is promoted as a learning activity within an organization that tolerates risk and does not shame and blame staff for evaluation failures (C), staff involved in evaluation are more apt to engage with and learn from evaluation (O) because they do not fear (M) disciplinary, punitive measures associated with evaluation failures [36–47].	
Examples	Supporting Quotes
*Example 1: Despite the intent of evaluation tools, staff may interpret them as ‘blame allocation devices’*	“We show also that the organisational participants charged with using the NHS-ST tool painted a different picture: these participants saw the NHS-ST primarily as a blame allocation device, informed by their previous experiences of performance management and accountability and by institutional features of the organisation of the NHS-ST programme. Ambiguities of measurement and widespread concerns about the tool's role in allocating responsibility for harm led to concerns about fairness. As a result, participants largely saw the NHS-ST as a way not of taking the temperature of their organisations and using it to improve care, but as a way of distributing heat – the potential for blame” [36].
*Example 2: When the motivating factor behind evaluation participation is avoiding blame and shame, fear becomes the driving force behind using evaluation as ‘proof’ of good performance, thereby suppressing learning*	“The evaluation is expected to generate necessary pressure for improvement through ‘naming and shaming’ effects… For local implementers, the pressure resulting from poor evaluation results is not inconsequential because ‘having a bad score not only makes you lose face’ in front of peers from all over the country, but may also create an impression [to local leaders] of your incompetence or lack of effort that can be detrimental to your career” [37].
*Example 3: Organizations that embrace risk facilitate learning from evaluation by focusing less on the evaluation outcomes and more on the potential opportunities for improvement that are exposed through evaluation*	“They’re interested right away… And then they really don’t mind when it’s not going well, as long as you know why it’s not going well. That is exactly what you want, then you’re going to use those numbers well” [48].
CMOC 2: Increasing Capacity for Evaluation When staff involved in evaluation receive leadership support, training, resources, and protected time and space to learn with others about the practical relevance of their data (C), they adopt evaluation work as part of their role (O) due to confidence (M) in their increased knowledge and abilities [38, 40, 41, 44, 46, 49–54].	
Examples	Supporting Quotes
*Example 1: Embedding learning into the evaluation process is enabled when staff have access to protected time and space to process and make sense of evaluation data*	“Quarterly QI project meetings were held on University premises, so that the internal project teams could take time out of the clinical setting to focus on the project… This enabled sites to learn from their own data and learn from each other’s experiences. These meetings enabled both internal project teams and external facilitators to identify problems in implementation and work towards overcoming them” [55].
*Example 2: Institutionalization of evaluation is enabled when linked levels of leadership are accountable for innovation performance reporting from front-line staff to senior leaders*	An informant described the ‘‘big focus of [departmental] leadership… on [guideline-related] quality indicators. It’s in their vision… and has an impact [on what happens on the unit]’’. The unit manager was required to formally report on guideline performance to her director” [50].
*Example 3: Resources to support evaluation may come in the form of changing the standard operating procedure of a ward or unit to include innovation and evaluation activities*	“Some respondents highlighted the importance of administrative reminders integrated in daily practice, such as checklists in patient files, to increase the awareness of and adherence to the ERAS programme [48].
Sequence 1 (CMOC 3 & 4)	
CMOC 3: Co-Producing Evaluation When evaluations are co-produced in an open context with transparency and meaningful inclusion of staff input (C) there is an increased likelihood that evaluation data will be used in decision-making (O) due to staff’s integral role in the evaluation process, their sense of ownership, and the increased value (M) they attribute to evaluation [14, 18, 26, 39, 42, 45, 51, 56–66].	
Examples	Supporting Quotes
*Example 1: Collaborative approaches to evaluation design facilitate engagement of staff and contextualization of data collected throughout the evaluation process*	“The CRE-IQI innovation platform concept and the [developmental evaluation] were developed and refined together drawing on input from multiple stakeholders and on purposeful opportunities to garner further Indigenous input. Placing importance on context, and valuing Indigenous knowledge by centring the voices of participant populations in the research, data analysis processes occurred collaboratively to capture a variety of worldviews that also embedded ‘member checking’ processes… This collaborative data analysis approach provided immediate, useable feedback to engage CRE-IQI stakeholders in co-creating solutions, thus reflecting some of the strong principles of [developmental evaluation]” [25].
*Example 2: Co-production can create an ‘internal locus of control’ (control over innovation and evaluation processes) for staff involved in the innovation and evaluation, thus increasing buy-in*	“The ability to influence every step in the process from the first step on, created time for discussion and reduced resistance among professionals. They valued the possibility to adapt the procedure step by step, which made it feel like their own initiative. When professionals are allowed to experiment and results are visible, chances that the new procedure is perceived as practical and useful increase… The information gathered during iterations, supported shared decision making about appropriate adaptations, which were then implemented step-wise and re-evaluated” [67].
*Example 3: Evaluation designs that do not include a mechanism for collecting contextual information including the concerns and realities of staff involved in the innovation may spur innovation or evaluation resistance*	“The equivocations that participants identified in determining what counted as a particular instance of a harm, its severity, and its recency could not be reported in the data themselves, which appeared tabulated in spreadsheet form devoid of contextual information Those in the sites were concerned that these data then became available for scrutiny, judgement, and blame, without any evidence remaining of the underlying local (and social) practices involved in producing them” [36].
*Example 4: Embedding evaluators within the innovation team enables real-time feedback to staff and leaders by those with a deep understanding of the innovation and its context*	“Consistent with a [developmental evaluation] approach, a research fellow, aka the [developmental evaluation] practitioner (JB), was embedded within the innovation platform team. This meant that any changes to its direction and evaluation – based on insights, learnings and critically reflective conversations between the evaluator and CRE-IQI management and members – could be facilitated rapidly as needs emerged” [25].
CMOC 4: Implementing Learning Feedback Loops When timely, evidence-based data are continuously assessed and collaboratively discussed between leaders and evaluators (C), the need for innovation improvements can be more effectively identified and prioritized within the organization by data users (O) because the evaluation data have meaningful (M) applications for them [18, 26, 39–42, 44–46, 48, 49, 56, 58–60, 68–75].	
Examples	Supporting Quotes
*Example 1: Reflection and responsiveness on the part of those involved in evaluation and those with the ability to take action based on evaluation are key attributes to embedding learning from evaluation data*	“Interview and documentary data clearly and consistently pointed towards one fundamental sustainability-oriented process that emerged over time. The strategy of “reflection and course-correction” meant that leaders continually assessed the “lessons learned” from their work on the program. Leaders then used these lessons to inform their modifications to the program. This was described as: “you never finish. We got to here, we’ve learned this, now let’s make it better. That is what helps in keeping [the program] alive. It’s evolving” [76].
*Example 2: Embedded evaluators can help in the real-time sense-making process of understanding relevant data captured from the clinical setting and transforming it into actionable practices for ongoing improvement*	“Because the process focused on utilisation, making sense of emergent findings involved the evaluator working with innovation platform participants to analyse and understand the data… The findings were further synthesised and prioritised during these interactions, and strategies to address them were identified through collaborative processes” [25].
*Example 3: Regular feedback to staff involved in evaluation of the benefits of the innovation to their patient populations can serve to stimulate innovation optimization and institutionalization*	“For example, we described aspects of institutionalization (i.e., regularity of performance monitoring activities) and of benefits (i.e., recognition of improved patient outcomes). In combination, these aspects prompted the adjustment of program goals, and ultimately contributed toward development (i.e., evolution of the program to achieve even further improvement)” [76].
Sequence 2 (CMOC 5 & 6)	
CMOC 5: Creating Sustainability Culture When evaluation data enable continuous refinements of innovations (C), staff sustain their evaluation and innovation efforts (O) because they are motivated (M) by the positive effects that evaluation has on improving innovations [36, 46, 47, 55, 66, 69, 77–79].	
Examples	Supporting Quotes
*Example 1: Evaluation can draw attention to important factors affecting the success of an innovation, thus enabling teams to target efforts toward priority challenges*	“As proposed by the programme theory, these individuals saw measurement through the NHS-ST as enabling problems that were previously occluded to become visible, thus facilitating assessment of size and scope of quality issues, and enabling identification of targets for action and monitoring of change” [36].
*Example 2: Knowledge use is contingent on understanding the interplay between innovations and context*	“Particularly within health care, it is vital to understand combinative capabilities and how knowledge is exploited to bridge the “know–do gap,” and implement research into reality… Even if studies have demonstrated unequivocal success, without considering factors of influence, it is impossible to gauge whether a health care system has the capacity to implement these evidence-based practices” [80].
*Example 3: Enabling the visibility of outcomes and integrating data collection and visualization techniques with institutional workflows and programs enables program reach*	“Audit and feedback of data, one of the most powerful influences on provider behavior, allowed continual refinement of the program. In addition, positive results, made available throughout the organization, created and sustained energy and commitment to the program” [79].
*Example 4: Evaluation is perceived as valuable when it helps to demonstrate the improvements being made by innovation efforts*	“Participants considered the availability and use of data to be a main driver of effective implementation as it allowed everyone to see concrete results of their efforts and allowed comparison to other centres… A majority of champions found that sharing the data reports helped overcome skepticism and resistance” [81].
CMOC 6: Becoming a Learning Organization When the organization internalizes lessons from evaluation by adapting practice and policies based on data (C), individuals within the organization experience a shift in norms, values, and beliefs toward that of a learning culture (O) due to attribution (M) of individual, team, and organizational learning as drivers of change [25, 26, 40, 41, 44, 47, 48, 67, 76, 80, 82–86].	
Examples	Supporting Quotes
*Example 1: Individual and team learning are linked through shared reflection on experiences*	“Including all professionals in the learning process requires special attention for individual and team learning during implementation of PSPs… To overcome resistance and improve adoption rates, extra time is needed to learn from experience and adapt PSPs to the local context. Experiential Learning (EL) emphasizes the value of successive learning cycles of end-users, where knowledge can be created and recreated through experience and reflection at individual and at team level. In these learning cycles, all professionals can be involved in the design and implementation process of the PSP, and make it effective in their own local work environment” [67].
*Example 2: Willingness on the part of the organization to shift and change in response to robust evaluation data enhances the sustainability of the innovation*	“…The full potential of a MFS is predicated upon the capacity (and willingness) of the practice organization to be disrupted by the innovative impact of its use… Continual monitoring of MFS fidelity and use is an essential part of the feedback loop back to the MFS developers to continually assess adaptations for impact on desired outcomes” [82].
*Example 3: Aligning and integrating clinical knowledge and intuition with evaluation data may help with sense-making and integrating evaluation practices into shared mindsets*	“A core feature of our implementation and training support was the valuing of multiple forms of evidence through the integration of quantitative data with the clinician’s intuition and experience” [82].
*Example 4: Evaluation activities may strengthen learning cultures through capacity-building and the creation of long-term learning processes*	“We observed that evaluating the innovation platform developmentally allowed for the acquisition of new knowledge and skills through multiple interactions with stakeholders. This ‘learning through knowledge exchange’ aligned well with one of the key elements of innovation platforms, which is “to enable long-term learning and capacity strengthening” and “knowledge generation and sharing” [4]. This design element went beyond co-creation because it emphasised the ongoing development of a learning culture” [25].

## Discussion

Each of the CMOCs is associated with specific Action Strategies to inform healthcare organizations’ leadership and/or the evaluative team. These Action Strategies are presented in Table 3. The following text includes references to literature that support these strategies by emphasizing their influence on the mechanisms of each CMOC.

**Table 3:** Strategies to action evaluation for learning and sustainability

Context	Mechanism	Associated Action Strategies
*Program Theory Domain: Organizational Preconditions*		
Tolerance of risk and failure in an organization	Staff’s fear of failure	*Embracing Risk & Failure* - Unite the leadership team in the goal of learning as the most important outcome of evaluation to avoid surface engagement and performative assessment by those involved in the work of innovation evaluation. This may mean getting comfortable with the fact that evaluation results may publicly expose organizational innovation challenges or failures - Establish the leadership team as a guiding coalition to support the evaluation by providing endorsement and visibility throughout the organization - Present the leadership’s united learning vision to staff involved in evaluation. Invite open dialogue about the tolerance of the leadership and organization to exposing innovation challenges and failures for the purposes of learning - Emphasize that evaluations are assessments of innovations, not individuals or teams - Celebrate failures as learning opportunities; do not associate failures with punitive measures
Protected time and space to learn with others	Staff’s confidence in their abilities	*Increasing Capacity for Evaluation* - Prepare for evaluation by ensuring the necessary time, training, and financial resources to support evaluation activities are in place before commencing evaluation - Establish evaluation governance and leadership teams with clear reporting structures and timelines. Enlist the governance and leadership teams to remove barriers for the evaluation team and staff involved in innovation - Position evaluation as part of the “daily work” of staff involved in innovation - In addition to evaluation as daily work, ensure focused time is protected for sharing learnings and reflections about evaluation on a regular basis - Continuously socialize new staff to evaluation work and provide refreshers of evaluation responsibilities on a regular basis to both new and existing staff
*Program Theory Domain: Sequence 1*		
Open and transparent contexts with meaningful inclusion of staff input	Feelings of value & ownership in staff	*Co-Producing Evaluation* - Collaborate with staff doing the work of innovation and evaluation to co-develop an evaluation logic model and make decisions about the data to be collected and its use - Regularly revisit the evaluation approach with those doing the work of innovation and evaluation (including staff, patients, families, and community partners) to ensure its continued fit with the ongoing innovation work - Encourage all staff in the innovation and evaluation teams to raise questions about the evaluation approach and suggest changes that may be necessary to make evaluation data more meaningful, and establish a process for incorporating feedback (from staff, patients, families, and community partners) into the evaluation approach on an ongoing basis - Position members of the evaluation team close to the work of innovation (such as embedded evaluators) to assist in both gathering relevant evaluation data, as well as making sense of learnings on an ongoing basis by those implementing the innovation through bidirectional knowledge exchange
Continuous assessment of data and collaborative data discussions between leaders and evaluators	Relevance to staff’s work	*Implementing Learning Feedback Loops* - Establish a plan for collecting, analyzing, reporting, and using data in collaboration with those with the power to take action based on the results of learnings generated from those data - Regularly assess to ensure the format and timing of data delivery continues to be useful for those with the power to take action on evaluation results - Establish a process for taking action to maintain, iterate, adjust, or stop the use of an innovation based on evaluation results - Establish a “feedforward” workflow for removing barriers to taking action on data in collaboration with organizational leaders, should it be needed
*Program Theory Domain: Sequence 2*		
Evaluation data enable continuous refinements of innovations	Staff’s observation of positive change	*Creating a Sustainability Culture* - Establish a process for taking action at individual, team, and organizational levels to translate learnings into actions for innovation sustainability - Continuously assess the fit of the innovation for the challenges it is being implemented to solve, based on ongoing learnings. Take action when evaluation data suggest that innovation refinements are needed - Recognize, collect, and communicate evidence of positive change as a result of innovation and evaluation often - Allow for evaluation to generate new questions or improvement opportunities on an ongoing basis by implementing a system to capture and prioritize new targets for action
Organization adapts practice and policies based on evaluation data	Staff’s observation of learning as a driver of change	*Becoming a Learning Organization* - Allow evaluation data to generate disruptions at the organizational level by building in flexibility and responsiveness into leadership teams, which can adapt to ongoing learning from evaluations - Value learning from praxis, intuition, and dialogue as well as learning from “intentionally collected” evaluation data - Establish knowledge-sharing forums where individuals and teams can share and reflect on their experience with innovations and their evaluations to integrate narratives into a collective experience - Intentionally connect individual, team, and organizational learning to change initiatives to demonstrate the impact of learning from evaluation on changes to practice and policies

The first CMOC emphasizes the importance of embracing reasonable risk and failure in evaluations to reduce fear and fuel learning and innovation sustainability. Anticipation and acceptance of unexpected outcomes of evaluation that resemble program failures can enable psychological safety in staff and foster deeper learning of the complexities of innovation [87]. To eliminate fear of failure and establish psychological safety in staff, it is important to engage in activities that unite the team, fuel open communication, and emphasize the value of learning from both successes and failures for the purpose of continuous learning and long-term innovation sustainability (Table 3). These actionable steps to diminish fear and establish psychological safety are supported in literature, where leadership teams that are united in the goal of learning from evaluation regardless of the outcome create a psychologically safe space for honest feedback [88, 89]. These collaborative and unified evaluation practices most often include having open within-team communication about evaluation purposes and goals, which further fosters psychological safety, trust, and openness to feedback [89–91]. To engage in collaborative evaluation and open communication, innovation failures revealed by evaluation should be celebrated as learning opportunities to reduce the fear of punishment [92] and enable innovation and improvement [93–95].

The second CMOC outlines the importance of increasing evaluation capacity to fuel learning and innovation sustainability by increasing staff’s confidence in their evaluation abilities. Strategies such as providing clear governance and appropriate data and human resources, establishing routine evaluation practices, and encouraging the development of evaluation skills in teams can build staff confidence in their evaluation abilities and increase evaluation capacity (Table 3). Providing clear structures and processes in organizations can provide staff with a framework for decision making and action, increase role clarity, and increase psychological safety [96–98]. Providing resources such as training and time signals to staff that evaluation is valued, and fosters a sense of capability in staff [96, 99–101]. The importance of protected time for shared learning, including shared learning of evaluation practices, can build staff’s confidence in their abilities as they can learn from their peers and troubleshoot challenges [87, 93]. Lastly, continuous onboarding and reinforcement of the responsibilities of new staff ensures staff are integrated in roles and fosters confidence in their knowledge and abilities, including their perceptions about evaluation as a central aspect of their role [100, 102, 103].

CMOC 3 discusses the importance of co-production in evaluation to fuel learning and innovation sustainability by establishing feelings of evaluation ownership in staff. Specifically, co-produced evaluation may foster learning and program sustainability, and generate high quality evaluations with relevant and applicable outcomes [33, 104, 105]. Strategies such as collaboration between innovation and evaluation staff, re-visiting evaluation approaches to assess ongoing relevance, and possible establishment of “embedded evaluators” should be considered as they can lead to increased staff feelings of ownership and value in the evaluations they produce, contributing toward evaluation and innovation sustainability (Table 3) [105–108]. Of note, co-production often includes the engagement of patient, family, and community partners in the healthcare context, yet in the literature that we reviewed, patient engagement was a clear gap. There is evidence to suggest that inclusion of patients, families, and community partners in all areas of LHS can lead to better health outcomes, more meaningful healthcare services for patients, and improved policy making [9]. The lack of involvement of patients, families, and community partners from the health innovation evaluation literature highlights the missed opportunities resulting from their exclusion from this important area of work. Future work should include patients, families, and community partners in evaluation of health innovations to realize the full benefits of embedding their expertise into learning health systems.

Implementation of learning feedback loops (CMOC 4) through collaborative data management, actioning evaluation results, and establishment of feedforward workflows [46] may increase the relevance of evaluations for staff. Collaborative evaluation practices such as joint data analysis meetings between evaluators and staff foster a deeper understanding of the evaluation findings, and increase the relevance and meaning of results for decision makers [107–109]. Feedforward loops provide future-oriented support to encourage improved organizational performance [110] by removing anticipated future barriers to evaluation and innovations and effectively acting on evaluation data [93, 110–112]. These collaborative data management and feedforward practices emphasize the relevance of staff’s evaluation work by using evaluation data to create positive change, thus supporting sustainable evaluation.

The final two CMOCs highlight the importance of the establishment of sustainability culture and learning organizations through staff’s observation of the positive changes produced as a result of learning through evaluation. Translating learnings into visible improvements, providing feedback on these improvements, and establishing a cycle of learning and improvement showcases the value of evaluation and provides staff with evidence of positive impact, fostering their motivation to continue evaluation and innovation [92, 107, 113]. Strategies that support the establishment of learning organizations are also supported by literature where integration of experiential (tacit) knowledge and explicit (data-driven) knowledge fosters staff participation in collaborative knowledge exchange [114–117]. Both the creation of a sustainability culture and establishment of a learning organization through action strategies that encourage staff observation of positive change and learning as a driver of change encourages continuous learning and innovation sustainability.

Finally, while many of the relationships explored in our CMOCs are derived from extant literature, future investigation should involve exploration of mid-range theories associated with the mechanisms of each CMOC. For instance, the CMOCs identified as Organizational Preconditions might relate to psychological safety where a culture of shame, blame, and an absence of risk tolerance may fuel fear in staff and may discourage them from viewing evaluation as a tool of learning and innovation sustainability [118]. CMOCs identified in Sequence 1 may relate to the role of evaluative inquiry where staff collaboration and cyclical learning from evaluations support the creation of learning organizations that view evaluation as a tool of learning and innovation sustainability [119]. Finally, Sequence 2 may relate to the pillars of the Dynamic Sustainability Framework (DSF) [18] where a sustainability culture and establishment of a learning organization may reflect the DSF’s emphasis on continuous problem solving for the purpose of improvement.

### Strengths and limitations

Due to the explosive growth in interest in health innovation sustainability from 2013 onward, we focused our review on the body of literature linking evaluation to learning and sustainability from the last decade. Additionally, we limited our screening of full text articles to the first 500 articles obtained for review efficiency. While these methodological decisions were validated with our Expert Committee and by checking our refined program theory for comprehensiveness and coherence, it is possible that inclusion of articles pre-dating 2013 or from outside of the first 500 full text articles would have changed the development of our refined program theory or CMOCs.

Strengths of this work included our ability to capture articles from a breadth of fields for inclusion in this work, adding to the generalizability and completeness of results. Additionally, the involvement of the Expert Committee in methodological and conceptual guidance throughout this work, and the diversity of expertise among members of this committee added to the rigour of results. Members of this Expert Committee included those with lived experience evaluating, developing and sustaining healthcare innovations, and leading healthcare organizations and influencing systems. It is through these combined lenses that guidance was sought and given for this work. Admittedly, a different group of experts with different experiences may have ultimately derived different results, but we are confident that the combined expertise of this group and the rigour with which this synthesis was conducted have yielded a valid and important contribution to our understanding of what makes evaluation ‘work’ to promote learning and sustainability of health innovations..

## Conclusion and recommendations

Through this realist synthesis, we found that organizational preconditions, as well as evaluation cycles that drive learning from health innovations, may over time shift organizations to establish a culture of innovation and evaluation sustainability. Our desire to translate findings into practical applications spurred the development of evidence-based Action Strategies associated with each phase of our program theory and CMOCs. Our results have implications for the Implementation Science community and those conducting evaluations of health innovations more broadly. Future research could include investigating mid-range theories connected to each identified mechanism and conducting field tests of the program theory and CMOCs by applying the action strategies and studying their effects on learning and health innovation sustainability.

## Supplementary Information

Supplementary Material 1.

Supplementary Material 2.

Supplementary Material 3.

## Data Availability

Requests for data beyond that publicly available in the manuscript and supporting files may be submitted to the corresponding author and will be available upon reasonable request.
