# Supplementary material for: Evaluating for learning and sustainability (ELS) framework: a realist synthesis
Source: BMC Health Serv Res. 2025 May 13;25:683. doi: 10.1186/s12913-025-12743-4 (PMC12070515; doi:10.1186/s12913-025-12743-4)
Supplement: Supplementary file 2 — Supplementary Material 2. [file 12913_2025_12743_MOESM2_ESM.pdf]

ELS Framework Additional File 2

Index

1. Data Sources
2. Search Process
3. Data Extraction

1. Data Sources

Primary databases searched

- MEDLINE
- Embase

Journals that were hand-searched (2013-2023)

- Implementation Science
- Learning Health Systems
- The Learning Organization
- BMC Health Services Research
- Evaluation & The Health Professions
- Evaluation

Websites that were hand-searched

- Agency for Healthcare Research and Quality (https://ahrq.gov/)
- The Learning Healthcare Project (https://learninghealthcareproject.org/)
- Nuffield Trust (https://www.nuffieldtrust.org.uk/)
- Alliance for Healthier Communities (https://www.allianceon.org/)

2. Search Process

Sample MEDLINE Search

Database: OVID Medline Epub Ahead of Print, In-Process & Other Non-Indexed Citations, Ovid MEDLINE(R) Daily and Ovid MEDLINE(R) 1946 to Present

November 16, 2022

Search Number	Reference Concept: Evaluation	Hits	Totals
1	Evaluation Study/ or Program Evaluation/	320426	
2	Qualitative Research/	77647	
3	Research Design/ or Nursing Research/	135335	
4	Evaluat*.tw,kf.	4208172	
5	((formative or summative or process or heuristic or developmental or framework) adj2 evaluat*).tw,kf.	22816	
6	(pilot or feasibility or questionnaire or survey or interview or usability or realist or pretest or pre-test or posttest or post-test or crossover or cohort or cross-sectional or crossover or trial or (case adj2 study) or (action adj2 research) or (focus ajd1 group) or quantitative or qualitative or (mixed adj1 method*) or usability or (use* adj2 test*) or RCT).tw,kf.	4199986	
7	((randomi$ed or control* or stepped or wedge or clinical or pragmatic or cluster) adj2 trial).tw,kf.	197949	
8	1 or 2 or 3 or 4 or 5 or 6 or 7		7531401
**Search Number**	**Reference Concept: Learning**	**Hits**	**Totals**
9	Learning/ or Quality Improvement/ or	148722	

	Organizational Culture/ or Organizational Innovation		
10	((Organi$ational or social) adj2 learning).tw,kf.	5366	
11	(Learn* adj2 (health* system)).tw,kf.	797	
12	((Double or triple) adj2 loop learning).tw,kf.	46	
13	((Meta or high) adj2 learning).tw,kf.	1450	
14	9 or 10 or 11 or 12 or 13		154816
**Search Number**	**Reference Concept: Sustainability**	**Hits**	**Totals**
15	(Sustainability or sustainment or scale or scale-up or spread or expand or expansion or dissemination or diffusion or extension).tw,kf.	1863126	
15	15		1863126
**Search Number**	**Reference Concept: Healthcare**	**Hits**	**Totals**
16	"Delivery of Health Care"/og [Organization & Administration]	22507	
17	Health or healthcare or (health ajd1 system) or clinic or (health adj2 service) or hospital or (primary adj1 care) or (emergency adj1 department) or (emergency adj1 room) or (doctor* adj1 office) or unit	4057805	
18	16 or 17		4064054
**19**	**8 and 14 and 15 and 18**		**3449**
**20**	**Limit: 2013-Current**		**2688**

3. Data Extraction

Data Extraction Table Operational Definitions

**Extraction Item**	**Explanation**
Central thesis	The main point(s) of the article. For a theoretical manuscript, the central thesis is the main tenet(s) of the theory or framework. For primary research, the central thesis is the main result or finding.
Foundations	Describe any theories or frameworks that the article builds upon.
Definitions	Pull out definitions offered by the article authors of important concepts.
Contexts	Contexts are factors that affect the resources, opportunities, or decisions available to individuals affected by evaluations. Contexts can operate at a very high level (e.g. health system/policy), down to the individual level (e.g. personal circumstances).
Mechanisms	Mechanisms are the underlying factors that drive the changes or outcomes observed as a result of evaluations.
Outcomes	Outcomes are the effects or changes observed as a result of evaluations.
Theory of Action	How the evaluation process "works" (i.e. how do changes in context spark mechanisms that drive outcomes?)
Disciplinary or epistemological assumptions	Any underlying or taken-for-granted "truths" that you come across in the articles. These could be as a result of the authors' profession, beliefs, or positioning.
Notes	Anything interesting/noteworthy that is not in another category.
