# Supplementary material for: Evaluating for learning and sustainability (ELS) framework: a realist synthesis
Source: BMC Health Serv Res. 2025 May 13;25:683. doi: 10.1186/s12913-025-12743-4 (PMC12070515; doi:10.1186/s12913-025-12743-4)
Supplement: Supplementary file 3 — Supplementary Material 3. [file 12913_2025_12743_MOESM3_ESM.pdf]

Draft Program Theory:

Evaluation helps to sustain innovation in a Learning Health System when it is undertaken in an enabling environment containing necessary and supportive resources, and when it drives an ongoing and cyclical learning process that helps to sustainably achieve outcomes.

Concept Clusters

The context of evaluation

1. Punishment vs. Learning

Evaluation is not neutral. The purpose of evaluative activities is to identify and scope "good" versus "poor" performance, for some overarching purpose. Depending on the interpretation of that purpose by those involved in implementing interventions, different reactions may be triggered (1). If the purpose of evaluation is perceived to be punitive or surveillant in nature, these individuals may be more likely to avoid and/or resist evaluation exercises, because they may fear that their individual or team performance will be scrutinized or punished (2). If, however, evaluative efforts are perceived by staff to be directed toward organizational learning with a mindset of innovation improvement, then staff may be more likely to embrace or engage in evaluation exercises, because they believe that they have a role in achieving these positive outcomes and do not fear punitive measures being taken as a result of evaluations (1, 2, 3). Individual-level factors that promote the perception of evaluative measures as learning-oriented include strong leadership (4), role modelling and positive reinforcement of candid information sharing (1, 2), and the demonstration of evaluation rigour, absence of bias, and respectful conduct (5). Organizational-level factors include fostering a culture of fairness and justice (1), embracing learning from failure (3), and rewarding staff for taking risks and trying new things (6).

2. Risk

If organizations are highly-risk averse, then they may be less willing to capture some forms of unpredictable data - such as emergent data (as opposed to indicators specified a priori) - as part of evaluations because they have less control over what happens after those data are collected and may not want to publicly acknowledge failures (7). The purpose of evaluation for risk-averse organizations may lean toward the identification and control of poor performance or poorly-performing individuals or teams (8). If organizations are more risk-tolerant, they may be open to collection, interpretation, and use of these unpredictable data because they recognize that these data represent an opportunity to learn (8). For risk-tolerant organizations, the purpose of evaluation may lean toward learning and value-creation, resulting in a climate that is more supportive of innovation (9).

3. Co-production

If innovations and evaluations are co-produced in an open context, with greater inclusive and transparency, then the perceived value and feasibility of the innovation is increased because people affected by the innovation were part of understanding the problem, as well as designing and testing solutions that fit the local context (10, 11, 12). Co-production also increases the trust that individuals have in the accuracy and meaningfulness of the data which ultimately assists in the creation of sustainable innovations because evaluation data are trusted and used to make evidence-informed decisions (12). In addition, specific aspects of co-production may foster and accelerate transformation, such as utilizing distributive leadership models to assist in sense-making and decrease the risk of leadership turnover (4) and fostering dialogue between innovation delivery and policy bodies to accelerate innovation penetration (13). However, co-produced innovations can also create challenges with regard to reconciling competing views and interests and establishing clear lines of accountability and rigour of the evaluation (14).

4. Evaluation education

If individuals are given ongoing and audience-specific education and training about evaluation they are more likely to stay engaged in evaluation activities because they recognize the organizational investment in them and their learning (1, 2, 9). *<u>This investment in evaluation education for individuals signals that evaluation is an organizational priority, increasing the likelihood of individual participation</u>*. Building capacity amongst local evaluators in interpreting routine data, synthesizing and applying evidence, and scaling up successful innovation are critical educational pursuits that contribute to innovation and evaluation sustainability (9). In addition, extending education beyond healthcare professionals to gear it toward patients and public citizens facilitates their engagement with knowledge generation and use (6, 9). Finally, providing foundational education on how evaluation tasks connect to organizational values and goals offers a chance for staff to connect their behaviours to the larger organization (15).

5. Individual and organizational learning

As an outcome of evaluation, learning at an individual level influences organizational learning because individuals contribute their knowledge gained from various sources to a shared learning ecosystem (1, 9). The life experiences of the individual, their tacit knowledge, exposure to and practice with learning from various sources, and their willingness and ability to contribute to the shared learning ecosystem will influence the learning that occurs as a result of evaluation at an organizational level (9). To this end, organizational structures must be in place to enable the development of a productive learning ecosystem for individuals, including organizational processes and procedures for knowledge contribution by individuals, constructive and nonpunitive efforts toward gathering all forms of knowledge and reducing errors (2, 9) and repositories for that information to be stored and used (9). Failure to harness knowledge at an individual level can create organizational susceptibility to “memory loss”, for example, when clinical teams rotate out of a setting and individual knowledge leaves with them (8). Learning at the individual and team levels can be facilitated by a focus on improvement rather than performance management (8, 10), group problem-solving and sense-making opportunities (1, 4, 16), and a focus on fostering trusting relationships conducive to informal knowledge sharing (1, 9). Organizational factors that contribute to learning include bidirectional feedback and feedforward links across organizational levels (9), routinized touchpoints for

reporting and responding to challenges (15), and mechanisms for collaboration between staff and executives for identification and removal of barriers to innovation success (17).

Evaluation framework development and planning

6. Value of evaluation

Evaluation efforts must be perceived as valuable to maintain engagement and support sustainability (8, 13, 14). Evaluation should be highlighted as an organizational priority, and the benefits of efforts expended by staff to conduct evaluation work and produce evaluation deliverables must be clearly and continuously articulated in feedback loops (3, 10, 11, 18). If staff can draw connections between their efforts directed toward evaluation and the effectiveness of those efforts, such as reduced system costs, improved system performance, patient care, team experience, individual success within an organization, or workflow efficiencies, their engagement with evaluation is likely to be sustained because they believe that their efforts are making a positive difference (1, 3, 13, 15, 18). If, however, evaluative efforts by staff are not perceived by staff to be valuable, for example, engagement with evaluation is conducted in a 'surface level' way, data collected or analyzed are not fed back to staff, or there is no perceived change or negative change in system performance, patient care, team experience, individual success within an organization, or workflow efficiencies, or innovations are perceived as a threat to staff, then staff are likely to disengage with evaluation activities because they cannot make the connection between their efforts and positive outcomes (6, 7, 19, 20).

7. Innovation lever

If evaluation serves as a lever to generate a productive evidence ecosystem to stimulate innovation, then evaluation is also a learning tool because it helps to shift the focus from practice and operational improvement to co-producing, sustaining, scaling, and spreading innovation to meet local needs in learning health systems (3, 4). Thus, evaluation leads to learning by linking the ability of an organization to both generate and apply practice, decision, and knowledge-related innovation data (4, 10). The quality of the data gathered through evaluation may support or hinder innovation— while complete and error-free datasets are optimal, even flawed, uncertain, proximate, or sparse data may be used to raise innovation and evaluation challenges and mobilize complex knowledge reflective of the context of innovation (19, 21). Further, data generated via implementation and use of an innovation (innofusion) can generate evaluation knowledge that can help to domesticate innovations and further improve fit for context (6). In times of disaster and upheaval, trends from routinely gathered data may serve as sentinels of change and assist organizations in pivoting to meet new and emerging demands by pursuing innovative solutions (22).

8. Choosing the right evaluation method

Evaluation can only act as a mechanism for learning and innovation sustainability if the evaluation is designed to capture the right outcomes, in the right way, from the right sources, and over the right time period to enable learning to take place (7, 14, 18, 23, 24, 25, 26). Careful scrutiny of evaluation frameworks for their fit with learning outcomes and an openness

to pragmatically designing evaluations to fit the desired learning outcomes is necessary to enable learning and sustainability outcomes (6, 10, 12, 18, 27). The design of these evaluations should be carefully considered, for example, the relevance of measurement markers to patients and clinicians, as well as their sensitivity to the 'fit' between innovation and context are key to achieving evaluation relevance (6, 10, 12, 25). Evaluations should capture implementation maturity, change in intended outcomes, as well as unintended outcomes and the mechanisms of action behind each— mixed or multi-methods evaluations, narrative storytelling, formative evaluations, or realist methods, rather than an over-reliance on purely quantitative studies and/or RCTs, may be better suited to achieving these objectives (3, 6, 7, 12, 18, 21, 24, 26, 27). Longitudinal monitoring and feedback, using a wide range of data sources, create the conditions for social acceptance and innovation sustainability (13, 18). In addition, evaluation design should consider the intended audience, time since innovation implementation and innovation maturity, and focus on process versus outcomes (18). A recognition that the most appropriate outcome measures may change over time in response to innovation evolution and contextual dynamism is essential (7, 28). Evaluation also has a role to play in creating space for dialogue, which enables learning through the process of evaluation as a socially constructed practice (16, 28). Evaluation designers may also want to consider building in a reflexive field note collection and review process to facilitate meaningful interpretation of results as well as to assist in anticipating and solving problems in real time (15). Finally, in order to utilize evaluations to benefit sustainability objectives, a parsimonious, comprehensive, and user-friendly monitoring tool with an explicit focus on both theoretical and practical sustainability requirements is required (14). The sustainability-focused evaluation must strike a balance between "rigour, rapidity, and flexibility of methods and procedures" (2), and the role of the evaluator should balance critical distance and immersion/engagement (28).

Learning cycles

9. Learning intention

For evaluation activities to produce learning, data generation, collection, analysis, reporting, and use must be implemented purposefully with the intention of producing learning (2, 7, 9). Conversely, if the data cycle is broken (e.g., data are collected but not analyzed or used to generate change where needed), evaluation will not lead to learning (29). If individuals, teams, and organizations habitually engage in full data cycles with the end point of utilizing knowledge gained to take action (including maintaining, iterating, adjusting, or stopping an intervention, according to the data), evaluation can produce learning by supplying evidence on which to make decisions (2, 4, 9, 10, 23). Crucial to being able to utilize data for learning is the ability to convert raw data to salient knowledge to help maximize innovation performance and health outcomes (3, 7). Various strategies can be used to operationalize learning cycles, such as implementing working groups and committees, community engagement strategies such as local health councils, organizational communities of practice, and facilitating experiential learning via practice and innovation labs (9). To maximize an organization's absorptive capacity for learning, these strategies should be undertaken in a learning-focused climate, which includes: leading with humility, valuing each individual, creating psychological safety, allocating sufficient time and resources for reflective thinking (16), and organizational resilience (6).

10. Timely feedback

In order to engrain the Learning Health System model, individuals must receive timely feedback about evaluation findings to continue to engage in the evaluation process (gathering, analyzing, utilizing data), and realize an appreciation of the utility of data to improve practice (1, 2, 7, 11, 15). The timing of evaluation feedback must be based on what is useful to the team doing the work of implementation and evaluation and should be flexibly adapted if needed (2, 5, 15, 17). Preliminary and emerging findings may be used by leadership teams in strategic planning and scale up processes (5), while analysis and feedback of long-term trends help to maintain front-line engagement over time (1).

11. Learning actualized in "use"

Learning is actualized in the "use" of knowledge gained being applied to practice, which serves to further inform individual and organizational learning, as well as optimize the innovation for sustainability (7, 10). Ongoing monitoring is key to this process, as it allows for evaluation to convince others of the value of the project; explore, investigate, and educate on what works, for whom, and why; demonstrate accountability in achieving objectives, create a living memory of the project, and engage individuals in a participatory process (14). Data derived from evaluation and monitoring activities may demonstrate whether practice changes are appropriate, effective, and efficient (23). Over time, these data can be linked to established and emerging organizational priorities, such that each enhances and engrains the other (8), helping to internalize the innovation to the fabric of organizational attitudes, norms, beliefs, and behaviours (1, 13). For long-term success, resources directed toward learning efforts and workflows should be balanced (2).

12. Visibility of learning

Evaluation modifies the way that we learn by making visible the processes of learning, which allows for scrutiny as to whether necessary components for learning are missing (9, 16). To engage in an authentic and collective learning process, organizations need to recognize and mitigate the perceived or real risks of exposing innovation dysfunction or poor performance (3). Pilot projects, in which a beta or test version of an innovation is implemented at a small scale, may assist in both allowing for and accepting failure at a smaller scale as part of the learning process, while enabling staff to build experience and expertise in the innovation, facilitating the likelihood of long-term success and sustainability (16). Pilot or full-scale innovation implementation may provide opportunities to learn about the innovation from sources such as information, deliberation, as well as praxis (9), in addition to learning about the context and the implications of contextual factors for realized outcomes (29). Creating strategic roles such as a field research coordinator can help to interpret outcomes in light of contextual factors, thus making learning visible and bridging the research-practice learning gap (15).

Innovation and evaluation sustainability

13. Continuity of evaluation

For evaluation to lead to sustainability, evaluation and learning activities must become engrained in workflows by adopting and adapting practice and policies that allow evaluation to become routinized (1, 4, 9, 10, 30). Continuous evaluation and learning allow for continual intervention adjustments to be made to adapt to changing context and maintain the intervention fit within dynamic circumstances over the long term (10, 12). The type of evaluation selected should be matched to the stage and maturity of the innovation (i.e., formative, process, summative, scale up, etc.), with the recognition that evaluation, rather than having a fixed end point, is in fact a central activity to innovation research and implementation, and should therefore be conducted continuously, using multiple, selected data collection periods (3, 12, 16). This longitudinal evaluation process should be designed flexibly to allow for iteration of hypotheses and indicators in light of emerging data (27). Conversely, short term evaluations or learning activities, such as efficacy trials which attempt to control for contextual variables extraneous to the intervention, will not necessarily contribute to intervention sustainability (10). Greater emphasis on achieving perfect fidelity to innovation delivery protocols with the belief that adherence will yield optimal results typically has the paradoxical effect of constraining necessary contextual adaptation, thus creating a mismatch between innovation and context and worsening outcomes (4, 10).

14. Resources for learning and sustainability

If people have an opportunity (time, resources) to review and reflect with their results/data then they will be more likely to be able to learn and sustain the entire evaluation/learning process (8, 16). Regular learning review activities that are routinized in individual, team, unit, or system workflows enhance the sustainability of the evaluation and learning process (1, 2, 8). Further, specific financial, technical, relational, and political resources have been identified as providing support for learning and innovation sustainability (13). Such resources include a devolved organizational structure, 'slack' resources that are able to be channeled into new projects (6, 8), comprehensive stakeholder engagement (1), capable and visible leadership, a risk-taking climate, opportunities for sense-making (2, 6, 8, 9, 20, 30), data accessibility and interoperability (3), and expedited processes for logistical aspects of innovation implementation such as ethical, legal, and human resourcing requirements (11). Individuals must also be given time and resources to allow for learning and the adaptation of the innovation to fit with local context (10, 13). Strategies to routinize reflexive evaluation practices include the use of running field notes to capture salient contextual variables, as well as regular dialogues between implementation and evaluation teams (15). A sustainability plan (1, 14) and distinct focus on sustainability in the planning phases of innovation development can help to actualize innovation sustainability (24).

References

1. Agency for Healthcare Research and Quality. Module 8: Organizational Learning and Sustainability. Communication and Optimal Resolution (CANDOR) Toolkit. Rockville: MD2016. p. https://www.ahrq.gov/patient-safety/settings/hospital/candor/modules/notes8.html.
2. Ellis LA, Sarkies M, Churruca K, Dammery G, Meulenbroeks I, Smith CL, et al. The Science of Learning Health Systems: Scoping Review of Empirical Research. JMIR Med Inform. 2022;10(2):e34907.
3. Labrique A, Vasudevan L, Weiss W, Wilson K. Establishing Standards to Evaluate the Impact of Integrating Digital Health into Health Systems. Glob Health Sci Pract. 2018;6(Suppl 1):S5-S17.
4. Steele Gray C. Overcoming Political Fragmentation: The Potential of Meso-Level Mechanisms Comment on "Integration or Fragmentation of Health Care? Examining Policies and Politics in a Belgian Case Study". Int J Health Policy Manag. 2022.
5. Desveaux L, Budhwani S, Stamenova V, Bhattacharyya O, Shaw J, Bhatia RS. Closing the Virtual Gap in Health Care: A Series of Case Studies Illustrating the Impact of Embedding Evaluation Alongside System Initiatives. J Med Internet Res. 2021;23(9):e25797.
6. Greenhalgh T, Wherton J, Papoutsi C, Lynch J, Hughes G, A'Court C, et al. Beyond Adoption: A New Framework for Theorizing and Evaluating Nonadoption, Abandonment, and Challenges to the Scale-Up, Spread, and Sustainability of Health and Care Technologies. J Med Internet Res. 2017;19(11):e367.
7. Foley T, Horwitz L, Zahran R. Realising the Potential of Learning Health Systems. Newcastle University, UK; 2021.
8. Reed JE, Howe C, Doyle C, Bell D. Simple rules for evidence translation in complex systems: A qualitative study. BMC Med. 2018;16(1):92.
9. Sheikh K, Abimbola S. Learning health systems: Pathways to progress. World Health Organization; 2021.
10. Chambers DA, Glasgow RE, Stange KC. The dynamic sustainability framework: addressing the paradox of sustainment amid ongoing change. Implement Sci. 2013;8:117.
11. Steels S, Ainsworth J, van Staa TP. Implementation of a "real-world" learning health system: Results from the evaluation of the Connected Health Cities programme. Learn Health Syst. 2021;5(2):e10224.
12. van Gemert-Pijnen JE, Nijland N, van Limburg M, Ossebaard HC, Kelders SM, Eysenbach G, et al. A holistic framework to improve the uptake and impact of eHealth technologies. J Med Internet Res. 2011;13(4):e111.
13. Cote-Boileau E, Denis JL, Callery B, Sabean M. The unpredictable journeys of spreading, sustaining and scaling healthcare innovations: a scoping review. Health Res Policy Syst. 2019;17(1):84.
14. Rey E, Laprise M, Lufkin S. Sustainability monitoring: Principles, challenges, and approaches. Neighbourhoods in Transition: The urban book series: Springer; 2022.
15. Reynolds J, DiLiberto D, Mangham-Jefferies L, Ansah EK, Lal S, Mbakilwa H, et al. The practice of 'doing' evaluation: lessons learned from nine complex intervention trials in action. Implement Sci. 2014;9:75.

16. Damschroder LJ, Aron DC, Keith RE, Kirsh SR, Alexander JA, Lowery JC. Fostering implementation of health services research findings into practice: a consolidated framework for advancing implementation science. Implement Sci. 2009;4:50.
17. Safaeinili N, Brown-Johnson C, Shaw JG, Mahoney M, Winget M. CFIR simplified: Pragmatic application of and adaptations to the Consolidated Framework for Implementation Research (CFIR) for evaluation of a patient-centered care transformation within a learning health system. Learn Health Syst. 2020;4(1):e10201.
18. Ovretveit J, Gustafson D. Evaluation of quality improvement programmes. Qual Saf Health Care. 2002;11(3):270-5.
19. Schlieter H, Marsch LA, Whitehouse D, Otto L, Londral AR, Teepe GW, et al. Scale-up of Digital Innovations in Health Care: Expert Commentary on Enablers and Barriers. J Med Internet Res. 2022;24(3):e24582.
20. Goldman J, Rotteau L, Flintoft V, Jeffs L, Baker GR. Measurement and Monitoring of Safety Framework: a qualitative study of implementation through a Canadian learning collaborative. BMJ Qual Saf. 2022.
21. Greenhalgh T, Papoutsi C. Studying complexity in health services research: desperately seeking an overdue paradigm shift. BMC Med. 2018;16(1):95.
22. Steele Gray C, Baker RG, Breton M, Kee K, Minkman M, Shaw J, et al. Will the “new” become the “normal”? Exploring sustainability of rapid health system transformations. 2021. In: Organising care in a time of COVID-19: Implications for leadership, governance, and policy [Internet]. Palgrave Macmillan Cham.
23. Rycroft-Malone J. The PARIHS framework--a framework for guiding the implementation of evidence-based practice. J Nurs Care Qual. 2004;19(4):297-304.
24. Shelton RC, Cooper BR, Stirman SW. The Sustainability of Evidence-Based Interventions and Practices in Public Health and Health Care. Annu Rev Public Health. 2018;39:55-76.
25. Proctor E, Luke D, Calhoun A, McMillen C, Brownson R, McCrary S, et al. Sustainability of evidence-based healthcare: research agenda, methodological advances, and infrastructure support. Implement Sci. 2015;10:88.
26. Bonten TN, Rauwerdink A, Wyatt JC, Kasteleyn MJ, Witkamp L, Riper H, et al. Online Guide for Electronic Health Evaluation Approaches: Systematic Scoping Review and Concept Mapping Study. J Med Internet Res. 2020;22(8):e17774.
27. Fletcher A, Jamal F, Moore G, Evans RE, Murphy S, Bonell C. Realist complex intervention science: Applying realist principles across all phases of the Medical Research Council framework for developing and evaluating complex interventions. Evaluation (Lond). 2016;22(3):286-303.
28. Greenhalgh T, Russell J. Why do evaluations of eHealth programs fail? An alternative set of guiding principles. PLoS Med. 2010;7(11):e1000360.
29. Abbott PA, Foster J, Marin Hde F, Dykes PC. Complexity and the science of implementation in health IT--knowledge gaps and future visions. Int J Med Inform. 2014;83(7):e12-22.
30. Swanson NM, Elgersma KM, McKechnie AC, McPherson PL, Bergeron MJ, Sommerness SA, et al. Encourage, Assess, Transition (EAT): A Quality Improvement Project Implementing a Direct Breastfeeding Protocol for Preterm Hospitalized Infants. Adv Neonatal Care. 2022.
